# Bile Acid Metabolites in Serum: Intraindividual Variation and Associations with Coronary Heart Disease, Metabolic Syndrome and Diabetes Mellitus

**DOI:** 10.1371/journal.pone.0025006

**Published:** 2011-11-14

**Authors:** Carine Steiner, Alaa Othman, Christoph H. Saely, Philipp Rein, Heinz Drexel, Arnold von Eckardstein, Katharina M. Rentsch

**Affiliations:** 1 Institute for Clinical Chemistry, University Hospital Zurich, Zurich, Switzerland; 2 Competence Center for Systems Physiology and Metabolic Diseases, ETH Zurich and University of Zurich, Zurich, Switzerland; 3 Center for Integrative Human Physiology, University of Zurich, Zurich, Switzerland; 4 Vorarlberg Institute for Vascular Investigation and Treatment (VIVIT), Feldkirch, Austria; 5 Department of Medicine and Cardiology, Academic Teaching Hospital Feldkirch, Feldkirch, Austria; 6 Private University of the Principality of Liechtenstein, Triesen, Liechtenstein; 7 Drexel University College of Medicine, Philadelphia, Pennsylvania, United States of America; Governmental Technical Research Centre of Finland, Finland

## Abstract

Bile acids (BAs) regulate glucose and lipid metabolism. In longitudinal and case-control-studies, we investigated the diurnal variation of serum concentrations of the 15 major BAs as well as the biosynthetic precursor 7α-hydroxy-4-cholesten-3-one (C4) and their associations, respectively, with coronary artery disease (CAD), diabetes mellitus type 2 (T2DM), and non-diabetic metabolic syndrome (MetS). In hourly taken blood samples of four healthy probands, the intraindividual 24 h variation of C4, conjugated and unconjugated BAs ranged from 42% to 72%, from 23% to 91%, and from 49% to 90%, respectively. Conjugated BA concentrations mainly increased following food intake. Serum levels of C4 and unconjugated BAs changed with daytime with maxima varying interindividually between 20h00 and 1h00 and between 3h00 and 8h00, respectively. Comparisons of data from 75 CAD patients with 75 CAD-free controls revealed no statistically significant association of CAD with BAs or C4. Comparisons of data from 50 controls free of T2DM or MetS, 50 MetS patients, and 50 T2DM patients revealed significantly increased fasting serum levels of C4 in patients with MetS and T2DM. Multiple regression analysis revealed body mass index (BMI) and plasma levels of triglycerides (TG) as independent determinants of C4 levels. Upon multivariate and principle component analyses the association of C4 with T2DM and/or MetS was not independent of or superior to the canonical MetS components. In conclusion, despite large intra- and interindividual variation, serum levels of C4,are significantly increased in patients with MetS and T2DM but confounded with BMI and TG.

## Introduction

The metabolic syndrome (MetS) is defined as a clustering of metabolic risk factors for cardiovascular disease including abdominal obesity, elevated blood pressure, impaired fasting glucose (IFG) or overt diabetes mellitus type 2 (T2DM), hypertriglyceridemia and low high density lipoprotein cholesterol (HDL-C) levels [1], [2], [3], [4]. In addition to these canonical components, patients with MetS frequently present with several additional homoeostatic disturbances in the regulation of metabolism, inflammation and coagulation, a state which has been termed metaflammation [5]. The symptoms and consequences of MetS are as heterogenous as the pathogenic origin appears to be diverse. Although excess or ectopic fat deposition and the resulting insulin resistance are considered as pivotal pathomechanisms, it is important to note additional possible pathogenic pathways. For example, disturbances in the intestinal microflora [6] and bile acid (BA) metabolism [7], [8] have been associated with MetS and T2DM.

The BA pool is constituted of primary BAs, which are synthesized in the liver by the classical and the alternative pathways, each involving a variety of different enzymes [9], as well as of secondary BAs, which are generated by deconjugation and/or dehydroxylation of primary BAs by intestinal bacteria. 7α-hydroxy-4-cholesten-3-one (C4) is a relatively sTable intermediate metabolite in the classical pathway of BA biosynthesis and is considered to be a plasma biomarker of BA synthesis [10]. Most human BAs are either conjugated to an amino acid, namely glycine (G) or taurine (T), or unconjugated. Primary BAs include cholic acid (CA) and chenodeoxycholic acid (CDCA) as well as their glycine- and taurine-conjugates (GCA, GCDCA, TCA, and TCDCA, respectively). Secondary BAs comprise deoxycholic acid (DCA), lithocholic acid (LCA) and ursodeoxycholic acid (UDCA) as well as their glycine- and taurine-conjugates (GDCA, GLCA, GUDCA, TDCA, TLCA, and TUDCA, respectively). After biliary secretion and intestinal deconjugation and/or dehydroxylation, BAs are extensively reabsorbed from the intestine and returned to the liver in order to be secreted again in the bile, thereby completing the enterohepatic circulation [11].

In the last decade, BAs were discovered to be natural ligands of the nuclear transcription factor farnesoid X receptor (FXR) [12], [13], [14]. In addition to their traditionally recognized role in cholesterol elimination and emulsification of dietary fat, BAs exert regulatory effects on their own biosynthesis but also on glucose and lipid metabolism via activation of FXR. The link between BAs and lipid metabolism was observed already in the 1970's when dyslipidemic patients treated with BA sequestrating resins, such as cholestyramine, were observed to present not only with decreased low density lipoprotein cholesterol (LDL-C) but also with increased plasma levels of triglycerides (TG) and HDL-C [15], [16], [17].

In vitro, BAs inhibit the production of very low density lipoproteins (VLDL) by cultured rat and human hepatocytes in a dose-dependent and BA species-dependant manner [18], [19]. Moreover, FXR knock-out (*Fxr^−/−^*) mice display elevated plasma levels of VLDL, low density lipoprotein (LDL) [20], and HDL-C [21]. Conversely, treatment of wild-type mice with a specific FXR agonist decreased plasma cholesterol levels. Underlying mechanisms include the repression of the transcription factor sterol regulatory element-binding protein 1c (SREBP-1c) and its lipogenic target genes [22], [23], as well as increased hepatic expression of receptors involved in lipoprotein clearance (VLDL receptor [24] and syndecan-1 [25]) and increased apoC-II levels (co-activates lipoprotein lipase) [26]. Taken together, these data suggest that FXR activation decreases plasma levels of all lipoprotein fractions. The effect of FXR activation on atherosclerosis is currently controversial. Despite their pro-atherogenic lipoprotein profile [20], *Fxr^−/−^* mice inconsistently presented with either increased or decreased atherosclerosis in different studies [27], [28], [29].

FXR also regulates gluconeogenesis, glycogen synthesis and insulin sensitivity. For example, hepatic glycogen levels were found to be increased in diabetic mice after FXR activation [30] and reduced in *Fxr^−/−^* mice [31]. In addition, activation or over-expression of FXR improved glucose tolerance and insulin sensitivity of diabetic mice [30], [32], whereas *Fxr^−/−^* mice showed peripheral insulin resistance and impaired glucose tolerance compared to wild-type mice [30], [32], [33]. These euglycemic effects of FXR activation are thought to be at least partly due to the repression of hepatic gluconeogenic genes (phosphoenolpyruvate carboxykinase (PEPCK) and glucose-6-phosphatase (G6Pase)) [30], [32]. However, in contrast to studies with diabetic mice, treatment of wild-type mice with FXR agonists has yielded inconsistent results on PEPCK activity or glucose levels [30], [33], [34], [35], [36]. Several studies have shown that the BA pattern is changed in experimental diabetic rats, with the CA pool being increased and the CDCA pool being decreased [37], [38], [39], [40].

The few and small studies characterizing the BA profile in patients with T2DM have yielded inconsistent results [41], [42], [43]. In this case-control study we explored whether coronary artery disease (CAD) or MetS and components thereof including T2DM are associated with different plasma concentrations of distinct BA species or of the BA precursor C4.

Due to the observation of a high interindividual variability, and because C4 and the BAs themselves are known to undergo diurnal variation in both rodents [44] and humans [45], [46], we studied the influence of daytime and prandial status on the serum concentrations of the 15 major human BAs as well as C4 in 4 healthy volunteers during 24 hours at 1 hour intervals.

## Materials and Methods

### Longitudinal study on intraindividual variation

Four healthy volunteers (2 males and 2 females) aged between 27 and 29 years with normal routine liver function tests were recruited from the staff of our institute to study BAs and C4 profiles over 24 hours. The study protocol was approved by the local ethics committee and written informed consent was obtained from all volunteers. An indwelling venous catheter was placed in the forearm of each subject. The first blood sample was drawn at 13h00 after all participants had taken an identical breakfast at 09h15 and an identical lunch at 11h30. During the overnight study, the participants had an identical supper and breakfast at 20h15 and 08h45, respectively. For practical reasons, blood from subject A was always drawn at the exact clock hour (xxh00), whereas blood from subject B, C and D was drawn with 10 min, 20 min and 30 min delay respectively compared to subject A. Starting at 13h00, 14 ml blood were drawn from each subject with 1 hour intervals during 24 hours. Serum was prepared by centrifugation of the whole blood at 3000 rpm for 10 min at 25°C after having allowed it to clot for at least 30 minutes. 500 µl serum aliquots were immediately frozen at −20°C until analysis.

### Case-control-study on association of BAs and C4 with CAD, T2DM and MetS

The study was approved by the Ethics Committee of the University of Innsbruck and all participants gave written informed consent. Six sex- and age-matched patient samples, each encompassing 25 patients were selected from a previously described cohort of consecutive patients undergoing coronary angiography for the evaluation of established or suspected sTable CAD [47]. The six patient groups differed by the presence or absence of CAD, nondiabetic MetS or T2DM: 1. CAD:no/MetS:no/T2DM:no; 2. CAD:yes/MetS:no/T2DM:no; 3. CAD:no/MetS:yes; 4. CAD:yes/MetS:yes; 5. CAD:no/T2DM:yes; 6. CAD:yes/T2DM:yes. In the CAD classification, the control group comprised cohorts 1+3+5 whereas the CAD patients group comprised cohorts 2+4+6. For the MetS/T2DM classification, the controls, the MetS patients and the T2DM patients comprised cohorts 1+2, 3+4, and 5+6 respectively. One patient from control cohort 1 (CAD:no/MetS:no/T2DM:no) presented with extremely high serum levels of BAs because of liver cirrhosis. Therefore his data were excluded from the statistical data analysis. No other patient presented with clinical manifestations of liver or bile duct diseases. However, asymptomatic liver disease cannot be ruled out since no abdominal ultrasound examination was performed. Liver enzyme acitivities were normal except for mildly elevated alkaline phosphatase activities in four patients who presented with 135 U/L, 139 UL, 183 U/L, or 212 U/L (upper reference range 129 U/L). None of the patients was treated with bile acid sequestrants.

According to NCEP-ATPIII guidelines, the MetS in individuals of cohorts 3 and 4 was diagnosed if three or more of the five following stigmata were present: waist circumference >102 cm in men and >88 cm in women, TG ≥1.7 mmol/l (150 mg/dl), HDL-C <1.0 mmol/l (40 mg/dl) in men and <1.3 mmol/l (50 mg/dl) in women, blood pressure ≥130/≥85 mmHg, and fasting glucose ≥6.1 mmol/l (110 mg/dl) but <7 mmol/L. T2DM in cohorts 5 and 6 was diagnosed by either fasting glucose levels ≥7 mmol/l (126 mg/dl), or plasma glucose levels ≥11.1 mmol/l (200 mg/dl) two hours after an oral bolus of 75 g glucose (i.e. 2 h OGTT) or previously diagnosed T2DM. “Metabolic controls” (cohorts 1+2) were defined by the absence of both MetS and T2DM.

Selective coronary angiography was performed by the Judkins technique. The angiograms were recorded in multiple projections with a biplanar digital cardiac imaging system (Philips Integris DCI). Cine angiograms were reviewed by experienced cardiologists who were blinded to DXA results. Stenoses were identified, and the percentage of lumen diameter stenoses was assessed by visual analysis. Significant CAD was diagnosed by the presence of significant coronary stenoses with lumen narrowing of at least 50% (cohorts 2, 4 and 6). In addition, the severity of CAD was quantified as the sum of all stenoses percentages of a given patient divided by the number of coronary stenoses in this patient. The extent was calculated as the number of significant coronary stenosis with lumen narrowing ≥50% in a given patient.

Venous blood samples were collected in the morning between 8h00 and 10h00 after an overnight fast of 12 hours and before angiography. Serum samples for BAs and C4 measurements were stored at −80°C.

### Analytical methods

All laboratory measurements except for BAs and C4 were performed on freshly isolated serum or plasma samples or full blood for glycated hemoglobin (HbA1c) in the central laboratory of the VIVIT study in Feldkirch (Austria). Serum levels of TG, total cholesterol, LDL-C and HDL-C were determined using enzymatic assays and precipitation techniques (TG: GPO-PAP, cholesterol: CHOD/PAP, LDL-C: QuantolipLDL, HDL-C: QuantolipHDL; all Roche, Basel, Switzerland) on a Hitachi-Analyzer 717 or 911. HbA1c was determined by high-performance liquid chromatography on a Menarini-Arkray KDK HA 8140 (Arkray KDK, Kyoto, Japan), and glucose levels were measured enzymatically from venous fluoride plasma by the hexokinase method (Roche, Basel, Switzerland) on a Hitachi 717 or 911. Insulin levels were determined by using an enzyme immunoassay on an AIA 1200 (Tosoh, Tokyo, Japan). The HOMA-insulin resistance index (HOMA-IR) was calculated according to Matthews et al. [48].

The 15 major human BAs (CA, CDCA, DCA, LCA, UDCA as well as their glycine- and taurine-conjugates) and the BA precursor C4 were quantified in two 100 µl-aliquots of serum using liquid chromatography-tandem mass spectrometry as previously developed by our group [49]. The limits of quantification (LOQ) ranged from 0.002 to 0.05 µmol/L for the different BAs and was 0.005 µmol/L for C4. Interassay imprecision and inaccuracy were both below 15% for every BA species and C4.

### Statistics

Statistical analyses were performed using IBM® SPSS® Statistics, version 19 (IBM Corporation, Somers NY, USA) and SIMCA-P+® 12.0.1.0 (Umetrics Inc., Umeå, Sweden). Values below LOQ were also included into the different statistical analyses.

Because data on BAs and C4 did not follow a Gaussian frequency distribution, univariate statistics were performed using the non-parametric Mann-Whitney and Kruskal-Wallis tests. A Bonferroni correction was used to compensate for multiple comparisons. Results were considered to be statistically significant when the p-value was below a threshold obtained by dividing 0.05 by the number of statistical tests per experiment, which corresponds to an α of 0.05. Correlations of BA parameters with each other and other continuous variables were calculated by using the Spearman rank test.

For multivariate and multiple regression analyses, data which did not follow a normal distribution were transformed into base 10 logarithms in order to obtain a frequency distribution close to Gaussian whenever possible. A multiple regression analysis with forward stepwise selection was used to identify independent contributors of MetS and T2DM components to BAs or C4 levels. Logistic regression with forward stepwise and backward stepwise selection was used to find any contributions of BAs or C4 to the classification into healthy or diseased adjusting for the commonly used biomarkers for MetS and T2DM as well as to calculate odds ratios.

An orthogonal partial least square-discriminant analysis (OPLS-DA) was used to estimate the importance of the individual variables as discriminating biomarkers. OPLS-DA was fitted in order for the classes to get the highest Q^2^ value. The confidence levels for the model parameters were set to 95% and the significance levels for DModX and Hotellings T2 were set to 0.05. Normalization of the distance to the model was achieved using standard deviation units. The SIMCA-P+® 12.0.1.0 program performed cross-validation with 7 groups as a default. CV-ANOVA (significance of Q2YCV using the F-distribution) was performed for the models as well as misclassification Tables and Fisher's probability. Data for diabetes medication as well as age and gender were not included in the model in order to avoid bias in the case of medication and because patients were matched for age and gender. Samples which were beyond the 95% confidence interval of the normal multivariate distribution (Hotelling's T2) were considered as outliers and were left out from the final model.

## Results

### Diurnal profiles of BAs and C4

**Figure 1:**
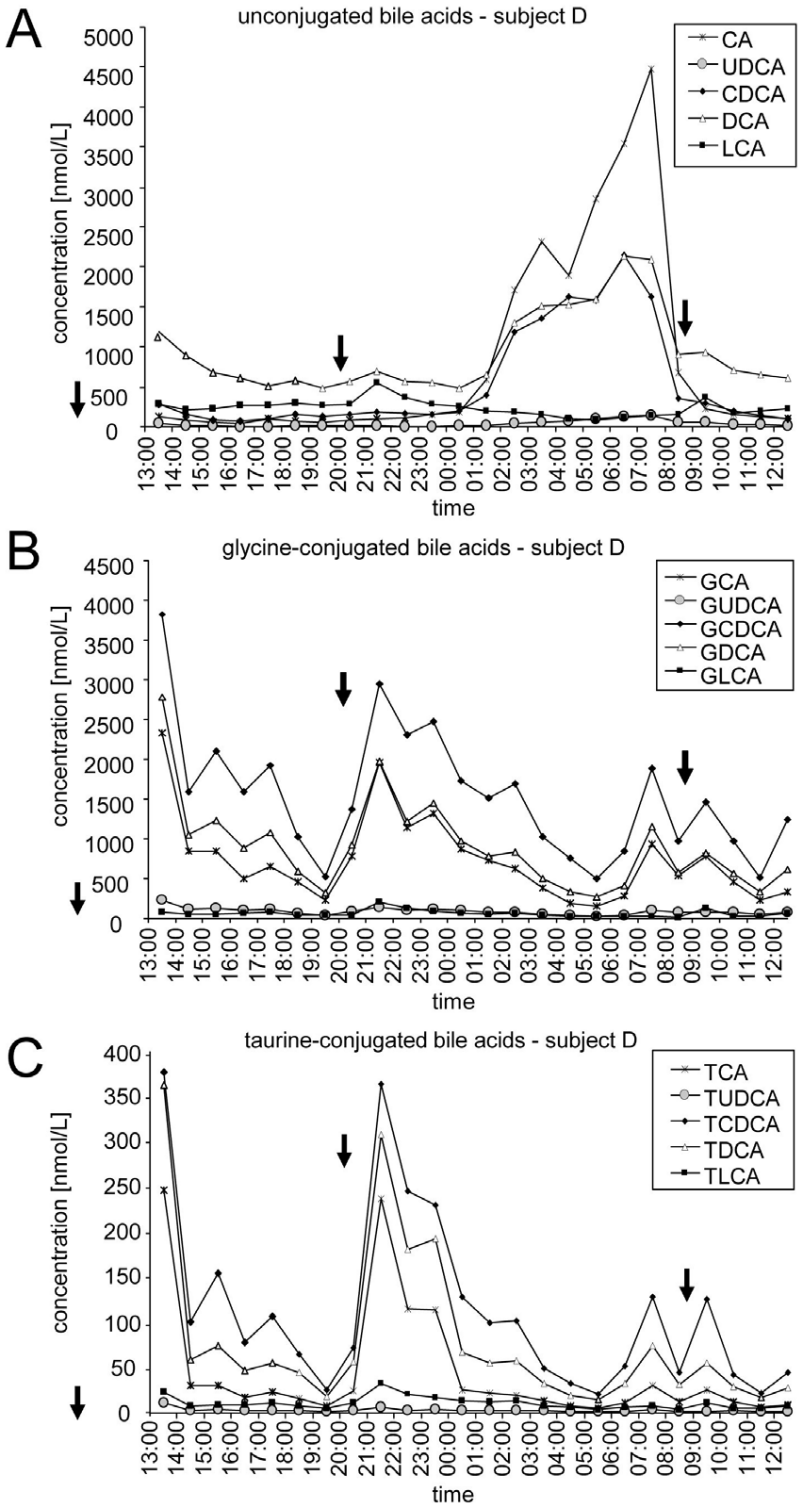
Intraindividual variations of individual unconjugated and conjugated bile acids during one day. Shown are diurnal profiles of individual unconjugated BAs (A), glycine-conjugated BAs (B), and taurine-conjugated BAs (C) concentrations in subject D. Meals are indicated with arrows. See text for abbreviations.

**Figure 2:**
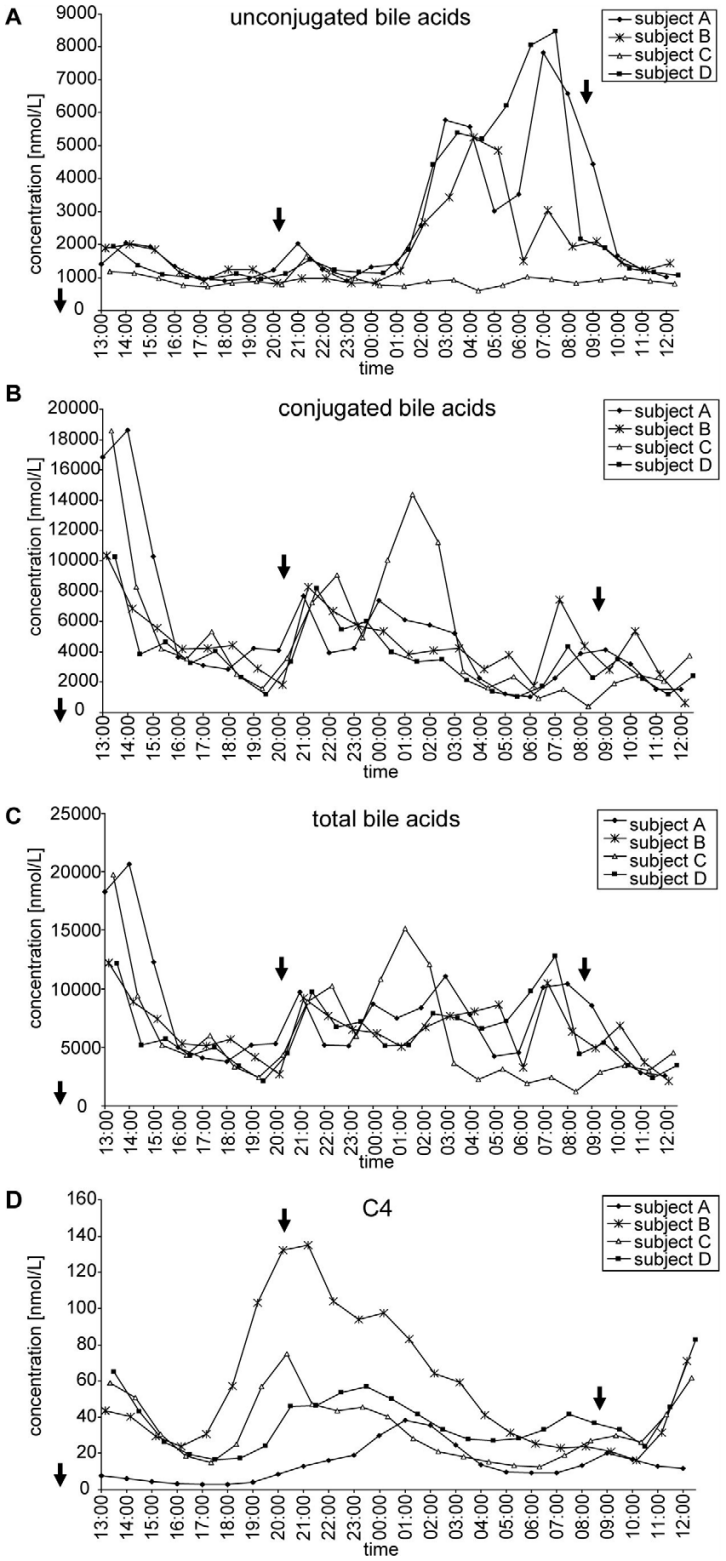
Intraindividual and interindividual variations of summarized unconjugated and conjugated bile acids during one day. Diurnal profiles of summarized unconjugated BAs (A), conjugated BAs (B), total BAs (C) and C4 (D) concentrations in all four subjects. Meals are indicated with arrows. See text for abbreviations.

Figure 1 shows representative diurnal profiles of individual unconjugated (Figure 1a), glycine-conjugated (Figure 1b) and taurine-conjugated BAs (Figure 1c). Figure 2 shows the summarized data of unconjugated (Figure 2a), conjugated (Figure 2b) and total BAs (Figure 2c) for the four subjects. Minimum and maximum concentrations as well as the intraindividual variability for the four subjects are shown in Table 1. According to mean values and standard deviations of measurements in 24 hourly taken blood samples, the intraindividual 24 h variation of conjugated and unconjugated BAs ranged from 23% to 91% and from 49% to 90%, respectively.

**Table 1:** Intraindividual variability of bile acids and C4.

Analyte	Conc_Min_	Conc_Max_	Subject A	Values <LOQ	Subject B	Values <LOQ	Subject C	Values <LOQ	Subject D	Values <LOQ
CA	<0.020–0.049	0.077–4.48	0.599±149%	1/24	0.372±155%	0/24	0.039±51%	5/24	0.828±154%	0/24
UDCA	<0.020	0.048–0.138	0.033±89%	10/24	0.009±147%	21/24	0.024±69%	11/24	0.037±106%	13/24
CDCA	0.039–0.163	0.641–2.41	0.489±129%	0/24	0.507±105%	0/24	0.322±31%	0/24	0.535±120%	0/24
DCA	0.271–0.480	0.770–2.96	0.983±63%	0/24	0.628±41%	0/24	0.488±22%	0/24	0.934±54%	0/24
LCA	<0.050–0.281	0.102–0.799	0.434±29%	0/24	0.350±48%	0/24	0.047±51%	14/24	0.234±43%	0/24
GCA	0.030–0.162	2.00–2.67	0.726±93%	0/24	0.788±55%	0/24	0.618±92%	0/24	0.733±73%	0/24
GUDCA	0.014–0.029	0.186–0.311	0.093±83%	0/24	0.077±48%	0/24	0.111±73%	0/24	0.088±48%	0/24
GCDCA	0.150–0.497	3.82–7.42	1.90±73%	0/24	2.34±47%	0/24	2.25±84%	0/24	1.53±52%	0/24
GDCA	0.080–0.415	2.01–5.61	1.75±77%	0/24	0.879±50%	0/24	0.976±91%	0/24	0.902±63%	0/24
GLCA	<0.050	<0.050–0.444	0.021±22%	24/24	0.164±63%	3/24	0.036±67%	18/24	0.059±72%	11/24
TCA	<0.005–0.015	0.136–0.700	0.108±167%	0/24	0.059±53%	0/24	0.168±95%	0/24	0.043±157%	2/24
TUDCA	<0.002	0.003–0.031	0.004±105%	8/24	0.001±70%	22/24	0.007±104%	5/24	0.002±122%	20/24
TCDCA	0.019–0.033	0.377–2.25	0.356±103%	0/24	0.183±58%	0/24	0.563±98%	0/24	0.112±89%	0/24
TDCA	0.010–0.022	0.161–1.88	0.235±190%	0/24	0.076±52%	0/24	0.432±107%	0/24	0.079±116%	0/24
TLCA	<0.005–0.005	0.031–0.069	0.021±62%	0/24	0.027±50%	0/24	0.015±66%	3/24	0.009±76%	7/24
C4	<0.005–0.016	0.038–0.135	0.014±72%	5/24	0.058±64%	0/24	0.034±51%	0/24	0.038±42%	0/24
Primary BAs	0.456–1.13	8.23–13.2	4.18±62%	-	4.25±43%	-	3.96±81%	-	3.78±54%	-
Secondary BAs	0.768–1.41	4.00–9.32	3.57±56%	-	2.21±34%	-	2.14±70%	-	2.34±37%	-
Unconjugated BAs	0.598–0.940	1.63–8.46	2.54±79%	-	1.87±65%	-	0.920±23%	-	2.57±92%	-
Conjugated BAs	0.386–1.03	10.2–18.6	5.21±85%	-	4.59±49%	-	5.18±89%	-	3.56±62%	-
Total BAs	1.22–2.55	12.2–20.7	7.75±59%	-	6.46±38%	-	6.10±77%	-	6.13±46%	-

Minimal and maximal concentrations and mean±variation coefficient are given for the 15 individual bile acids, C4 and the grouped bile acids for all four subjects. Concentrations are given in [µmol/L] and intraindividual variability is given as the mean concentration [µmol/L]±CV [%].

The profiles of unconjugated BAs differed markedly from the profiles of the glycine- and taurine-conjugated BAs. In three out of four subjects, we observed a strong increase of unconjugated BAs during the night and early morning, which actually seemed to consist of two different peaks (Figures 1a and 2a). These peaks were mainly caused by CA, CDCA and DCA which reached maximal concentrations of about 4.5 µmol/L, 2 µmol/L, and 2 µmol/L, respectively (Figure 1a). In subjects A and B, levels of CA reached maximal concentrations of 3 µmol/L and 2 µmol/L, respectively (data not shown). The acrophase lasted from approximately 01h00 to 08h00 in subjects B and D and from 02h00 to 10h00 in subject A. Subject C however showed a different profile with a much less intense peak at approximately 21h00 (CDCA and DCA). In order to disclose a potential dysregulation of the circadian clocks of subject C, cortisol concentrations in serum were measured and it was found that all 4 subjects showed expected cortisol profiles with acrophases in the morning between 06h00 and 11h00 (data not shown).

By contrast to unconjugated BAs, conjugated BA levels seem to depend strongly on food intake since both lunch and supper were followed by increases in the concentrations of conjugated BA (increased concentrations before 15h00 following the meal at 11h30 and between 20h00 and 00h00 following the meal at 20h15) (Figures 1b and 2b). It is also important to note that all glycine and taurine conjugated BA species followed the same rhythm, although with different absolute concentrations. The increase after breakfast at 08h45 was less clear and the concentrations had already started to increase at 07h00 before the meal was taken at 08h45.

Due to fluctuations of unconjugated and conjugated BAs, the total BA concentrations also vary considerably throughout the day. Their profile was mainly influenced by conjugated BAs and, hence, food intake (Figure 2c).

Figure 2D shows the diurnal variation of C4 in the four volunteers. C4 levels show a similar degree of intraindividual variation like BA's ranging between 42% and 72% (Table 1). Despite considerable differences between the individual profiles, both diurnal and prandial effects appear to affect C4 levels: The highest C4- levels were seen between 19h00 and 1h00, the lowest C4 levels between 5h00 and 7h00 as well as between 16h00 and 18h00. In addition, the data do not rule out a slight postprandial increase of C4 levels which may explain the moderate acrophase around noon (Figure 2D).

### Case-control studies

In order to simplify the initial analysis and to avoid excessive multiple testing, the 15 individual BAs were grouped into primary BAs (sum of CA, CDCA, GCA, GCDCA, TCA and TCDCA) versus secondary BAs (sum of DCA, LCA, UDCA, GDCA, GLCA, GUDCA, TDCA, TLCA and TUDCA), unconjugated versus conjugated BAs, and total BAs (sum of all 15 BAs). The BA precursor C4 was analyzed individually. In a first step we tested whether age or gender affects BA or C4 concentrations. Neither the 5 BA groups nor C4 were affected by age or gender, neither in the healthy cohort 1 (CAD:no/MetS:no/T2DM:no) nor in the entire cohort of 149 individuals (Table S1 and Table S2). Furthermore, there were no differences in levels of the 5 BA groups or C4 between treated versus untreated patients for statin use, insulin therapy or combined oral hypoglycemic drugs (Table S2). We therefore performed further statistical analyses on unadjusted data.

### Associations of CAD status with BAs and C4 levels

Table 2 compares the demographic, anthropometric and clinical data of 75 patients with CAD and 74 controls free of significant CAD. The two groups differed significantly from each other only by systolic blood pressure, the prevalence of statin treatment (35% versus 59%) as well as the severity and extent of CAD. Table 3 describes the medians and ranges for the 5 BA groups and C4 in CAD patients and CAD free controls. Mann-Whitney tests revealed no statistically significant differences in plasma levels of C4 or grouped BAs between CAD free controls and CAD patients. This was also true when comparing the data of the following cohorts: CAD:no/MetS:no/T2DM:no versus CAD:yes/MetS:no/T2DM:no; CAD:no/MetS:yes versus CAD:yes/MetS:yes and CAD:no/T2DM:yes versus CAD:yes/T2DM:yes. Also post-hoc analyses of individual bile acid species did not reveal any statistically significant difference between the two groups. We therefore performed the subsequent comparisons on the associations of BAs and C4 with MetS and T2DM without further sub-stratification for the presence of absence of CAD.

**Table 2:** Baseline characteristics of the study population according to CAD classification.

	CAD free controls	CAD patients	p-value^a^
	median (range)	median (range)	controls vs CAD patients
Number of subjects	74	75	-
Female/Male	44/30	45/30	0.946
Age	60 (52–73)	61 (55–72)	0.494
BMI [kg/m^2^]	29.7 (20.9–40.9)	29.0 (19.1–51.8)	0.235
Waist circumference [cm]	105 (75–136)	103 (73–144)	0.834
Systolic pressure [mmHg]	130 (105–180)	135 (110–165)	0.039
Diastolic pressure [mmHg]	80 (60–110)	80 (60–100)	0.923
Glucose [mM]	5.80 (4.22–19.20)	5.77 (4.44–16.43)	0.631
HbA1c [%]	5.9 (4.9–13.6)	5.8 (5.0–10.1)	0.893
Cholesterol [mM]	5.37 (2.59–8.39)	4.77 (1.94–8.44)	0.064
LDL-cholesterol [mM]	3.25 (1.14–6.29)	3.03 (0.67–6.24)	0.167
HDL-cholesterol [mM]	1.30 (0.54–4.35)	1.22 (0.67–2.23)	0.322
Non HDL-cholesterol [mM]	3.79 (1.50–7.02)	3.42 (0.93–6.37)	0.192
TG [mM]	1.69 (0.41–5.91)	1.51 (0.61–5.62)	0.333
AST [U/L]	26 (14–140)	26 (14–61)	0.797
ALT [U/L]	29 (10–136)	28 (15–133)	0.967
Estimated GFR (Mayo quadratic formula)	97 (61–123)	96 (40–119)	0.989
HOMA IR*	3.20 (0.53–47.14)	2.93 (0.21–26.49)	0.478
Smoking (no/yes)	29/45	22/53	0.205
Statins (no/yes)	48/26	31/44	0.004
Oral antidiabetic drugs (no/yes)	62/12	61/14	0.694
Insulin (no/yes)	67/7	67/8	0.807
Severity CAD	-	80.0 (42.5–130.0)	-
Extent CAD	-	2 (1–8)	-

a The Mann-Whitney U test was used to test quantitative variables and the Chi-square test was used to test qualitative variables.

*8 missing values (4 in CAD free controls and 4 in CAD patients).

**Table 3:** Associations of bile acids and C4 levels with CAD.

	CAD free controls	CAD patients	p-value^a^
	median (range) [µmol/L]	median (range) [µmol/L]	controls vs CAD patients
C4	0.047 (0.006–0.260)	0.033 (0.003–0.266)	0.167
Primary bile acids	1.73 (0.154–13.0)	1.44 (0.207–9.27)	0.245
Secondary bile acids	1.33 (0.073–8.93)	1.03 (0.073–5.30)	0.051
Unconjugated bile acids	1.19 (0.112–8.20)	1.02 (0.165–8.26)	0.247
Conjugated bile acids	1.32 (0.115–21.1)	1.16 (0.221–6.50)	0.289
Total bile acids	3.29 (0.227–22.0)	2.53 (0.712–9.40)	0.113

In order to account for multiple comparisons, a Bonferroni-corrected α error of 0.0083 (6 tests) was set as significance threshold for the Mann-Whitney test.

a Mann-Whitney U test.

### Associations of MetS or T2DM with BAs and C4 levels

Table 4 compares the demographic, anthropometric and clinical data of cohorts which had either T2DM (N = 50), non-diabetic MetS (N = 50) or were free of either metabolic disorder (N = 49). As expected by the definitions of MetS and T2DM, both diseased cohorts differed significantly from healthy controls by body mass index (BMI), waist circumference, systolic blood pressure, HbA1c, HDL-C, TG, and HOMA-IR index. In addition, T2DM patients and MetS patients differed from each other by waist circumference, glucose, HbA1c, HOMA-IR index, TG, total cholesterol, non-HDL-C and LDL-C as well as by treatment with statins, oral antidiabetics, and insulin.

**Table 4:** Baseline characteristics of the study population according to MetS and T2DM classification.

	MetS and T2DM free controls	MetS patients	T2DM patients	p-value^a^	p-value^a^	p-value^a^
	median (range)	median (range)	median (range)	controls vs MetS	controls vs T2DM	MetS vs T2DM
Number of subjects	49	50	50	-	-	-
Female/Male	29/20	30/20	30/20	0.934	0.934	1.000
Age	61 (55–68)	61 (52–73)	61 (54–72)	0.520	0.836	0.584
BMI [kg/m^2^]	27.4 (19.1–42.1)	29.6 (21.8–37.7)	31.2 (20.0–51.8)	0.005	1.33E-04	0.055
Waist circumference [cm]	97 (73–135)	105 (86–128)	110 (83–144)	2.63E-04	1.79E-05	0.046
Systolic pressure [mmHg]	130 (105–165)	135 (110–180)	140 (110–165)	0.019	0.001	0.200
Diastolic pressure [mmHg]	80 (60–100)	85 (65–110)	80 (60–100)	0.042	0.242	0.372
Glucose [mM]	5.38 (4.22–6.27)	5.58 (4.61–6.72)	8.05 (5.16–19.20)	0.069	6.47E-15	8.31E-14
HbA1c [%]	5.6 (4.9–6.1)	5.8 (5.3–6.4)	7.4 (5.9–13.6)	0.010	5.65E-17	1.38E-15
Cholesterol [mM]	5.39 (2.59–7.80)	5.39 (3.13–8.44)	4.48 (1.94–8.31)	0.559	0.026	0.002
LDL-cholesterol [mM]	3.13 (1.48–5.46)	3.56 (1.14–6.24)	2.60 (0.67–6.29)	0.153	0.009	2.55E-04
HDL-cholesterol [mM]	1.53 (0.98–4.35)	1.10 (0.54–2.07)	1.20 (0.67–2.12)	5.00E-09	1.23E-04	0.324
Non HDL-cholesterol [mM]	3.42 (1.50–6.01)	4.22 (2.25–7.02)	2.90 (0.93–6.76)	4.97E-04	0.206	1.41E-04
TG [mM]	1.10 (0.41–5.62)	2.12 (1.07–4.59)	1.65 (0.62–5.91)	1.41E-12	5.88E-05	0.014
AST [U/L]	26 (15–49)	27 (14–140)	25 (14–55)	0.651	0.338	0.190
ALT [U/L]	26 (14–82)	31 (10–136)	29 (12–133)	0.011	0.069	0.539
Estimated GFR (Mayo quadratic formula)	95 (63–120)	97 (61–123)	97 (40–120)	0.705	0.657	0.937
HOMA IR*	2.11 (0.21–8.96)	2.95 (0.87–26.31)	6.35 (1.11–47.14)	0.002	1.38E-09	1.03E-05
Smoking (no/yes)	16/33	19/31	16/34	0.578	0.945	0.529
Statins (no/yes)	32/17	30/20	17/33	0.585	0.002	0.009
Oral antidiabetic drugs (no/yes)	49/0	49/1	25/25	0.320	1.03E-08	4.46E-08
Insulin (no/yes)	49/0	50/0	35/15	-	3.15E-05	2.66E-05

a The Mann-Whitney U test was used to test quantitative variables and the Chi-square test was used to test qualitative variables.

*8 missing values (4 in MetS and T2DM free controls, 2 in MetS patients and 2 in T2DM patients).

Table 5 summarizes the medians and ranges for the 5 BA groups and C4 in the three subgroups. Only C4 levels showed a statistically significant difference as reported by the Kruskal Wallis test, which however was lost after correction for multiple comparisons (p = 0.009; α = 0.05 corresponds to p = 0.0083). Separate comparisons of the data from MetS patients and T2DM patients with those of controls by Mann-Whitney tests revealed a statistically significant difference in C4 levels between controls and T2DM patients, which persisted also after Bonferroni correction for multiple comparisons (p = 0.002; α = 0.05 corresponds to p = 0.0028). None of the other BA parameters showed any difference between the three groups. Neither did the post-hoc analyses of individual bile acids reveal any statistically significant difference between the three groups.

**Table 5:** Associations of bile acids and C4 levels with MetS and T2DM.

	MetS and T2DM free controls	MetS patients	T2DM patients	p-value^a^	p-value^b^	p-value^b^	p-value^b^
	median (range) [µmol/L]	median (range) [µmol/L]	median (range) [µmol/L]	controls vs MetS vs T2DM	controls vs MetS	controls vs T2DM	MetS vs T2DM
C4	0.029 (0.004–0.146)	0.054 (0.006–0.258)	0.052 (0.003–0.266)	0.009	0.043	0.002*	0.420
Primary bile acids	1.75 (0.154–13.0)	1.29 (0.299–9.56)	1.67 (0.207–9.27)	0.485	0.243	0.711	0.416
Secondary bile acids	1.10 (0.073–8.93)	0.996 (0.241–8.01)	1.33 (0.115–5.30)	0.290	0.834	0.210	0.150
Unconjugated bile acids	0.999 (0.112–8.20)	1.13 (0.287–5.52)	1.28 (0.195–8.26)	0.329	0.429	0.138	0.478
Conjugated bile acids	1.46 (0.115–21.1)	0.928 (0.342–16.4)	1.18 (0.172–6.50)	0.496	0.210	0.716	0.517
Total bile acids	2.85 (0.227–22.0)	2.40 (0.884–17.6)	3.33 (0.831–9.73)	0.409	0.467	0.524	0.188

In order to account for multiple comparisons, a Bonferroni-corrected α error of 0.0083 (6 tests) was set as significance threshold for the Kruskal-Wallis test whereas a corrected α error of 0.0028 (18 tests) was set as significance threshold for the Mann-Whitney test.

a Kruskal-Wallis test.

b Mann-Whitney U test.

*indicates a statistically significant difference after Bonferroni correction for multiple testing.

Because all T2DM patients also fulfilled the definition of MetS, we also compared the compiled data of these two patient groups (i.e. T2DM+MetS) with those of the controls. In this analysis also, serum levels of C4 differed significantly from those of controls (controls: median = 0.029 µmol/L, range = 0.004–0.146 µmol/L; combined MetS and T2DM: median = 0.054 µmol/L, range = 0.003–0.266 µmol/L; p = 0.003). No difference was observed between controls and combined MetS and T2DM patients for any of the BAs.

Because of previous reports we also tested whether MetS and T2DM patients differ from controls by the ratio of primary to secondary BAs [50], the glycine-conjugated BA GCDCA [51] or the ratio of CA to CDCA [43]. The ratio of primary to secondary BAs differed significantly between diabetics and controls (p = 0.011), but this statistical significance was lost after correction for multiple comparisons. When we compared the compiled data of the MetS and the T2DM group with controls, the ratio primary/secondary BAs also differed between patients and controls (p = 0.042), but again the statistical significance was lost after correction for multiple comparisons.

### Correlations of C4 and BAs

Table 6 shows the bivariate correlations of C4 and grouped BAs with each other as well as with biomarkers of MetS or T2DM. C4 plasma levels showed weak but statistically significant correlations with unconjugated BAs (r = 0.199; p = 0.015) secondary BAs (r = 0.210; p = 0.010) and total BAs (r = 0.165; p = 0.044). C4 correlated more with some components of the MetS, glucose and lipid metabolism, namely with BMI (r = 0.210; p = 0.010), glucose (r = 0.174; p = 0.034), HbA1c (r = 0.271; p = 0.001), HOMA-IR (r = 0.317; p = 1.29E-04), TG (r = 0.356; p = 8.35E-06), non-HDL-C (r = 0.260; p = 0.001), total cholesterol (r = 0.219; p = 0.007) and LDL-C (r = 0.213; p = 0.009).

**Table 6:** Spearman correlation matrix (2-tailed) of bile acids and other continuous variables.

Primary BAs	0.088																						
Secondary BAs	0.210*	0.518**																					
Unconjugated BAs	0.199*	0.658**	0.591**																				
Conjugated BAs	0.057	0.735**	0.670**	0.297**																			
Total BAs	0.165*	0.915**	0.773**	0.773**	0.774**																		
Primary / secondary BAs	-0.146	0.527**	-0.377**	0.088	0.156	0.187*																	
age	0.071	-0.028	-0.006	-0.030	0.022	0.006	-0.022																
BMI	0.210*	0.049	-0.054	0.096	-0.021	0.042	0.095	-0.027															
systolic BP	0.078	-0.060	-0.060	0.111	-0.154	-0.043	-0.043	0.090	0.124														
diastolic BP	0.055	-0.006	-0.068	0.091	-0.120	-0.022	-0.008	-0.070	0.135	0.569**													
glucose	0.174*	0.102	0.111	0.109	0.052	0.137	-0.074	0.086	0.349**	0.267**	0.154												
HbA1c	0.271**	0.095	0.166*	0.203*	0.016	0.168*	-0.104	-0.095	0.324**	0.257**	0.084	0.736**											
ASAT	-0.077	0.023	-0.017	-0.103	0.141	-0.003	0.042	-0.001	-0.006	-0.130	-0.021	-0.041	-0.090										
ALAT	0.087	0.045	0.004	0.015	0.102	0.023	0.020	-0.113	0.154	0.008	0.150	0.136	0.142	0.742**									
GFR mayo	-0.046	0.048	0.044	0.048	0.076	0.028	-0.006	-0.419**	0.103	0.034	0.003	0.002	0.032	0.080	0.285**								
total cholesterol	0.219**	0.084	0.053	0.105	0.038	0.076	0.035	0.011	-0.090	-0.061	-0.007	-0.262**	-0.207*	-0.068	-0.121	0.010							
LDL-cholesterol	0.213**	0.064	0.027	0.105	-0.002	0.053	0.054	0.000	-0.021	-0.024	0.003	-0.287**	-0.244**	-0.070	-0.124	0.039	0.908**						
HDL-cholesterol	0.017	-0.048	-0.073	-0.086	-0.033	-0.055	0.012	0.006	-0.286**	-0.059	-0.045	-0.220**	-0.141	-0.167*	-0.228**	-0.142	0.334**	0.189*					
non-HDL-cholesterol	0.260**	0.083	0.076	0.131	0.046	0.085	0.022	0.016	0.007	-0.014	0.028	-0.210*	-0.174*	0.007	-0.024	0.069	0.917**	0.918**	-0.001				
triglycerides	0.356**	0.132	0.181*	0.201*	0.100	0.177*	-0.057	-0.065	0.181*	0.071	0.187*	0.183*	0.213**	0.050	0.165*	0.161*	0.359**	0.295**	-0.420**	0.560**			
waist circumference	0.127	0.014	-0.031	0.121	-0.042	0.023	0.018	-0.045	0.832**	0.196*	0.165*	0.379**	0.325**	-0.037	0.152	0.179*	-0.113	-0.052	-0.286**	-0.012	0.210*		
HOMA-IR	0.317**	0.141	0.119	0.223**	0.072	0.173*	-0.014	-0.050	0.498**	0.188*	0.169*	0.649**	0.604**	-0.017	0.202*	0.129	-0.201*	-0.183*	-0.265**	-0.130	0.238**	0.486**	
severity CAD	-0.108	-0.190	0.016	-0.014	-0.068	-0.104	-0.223	0.001	0.185	0.015	-0.075	0.082	0.010	-0.030	-0.029	0.207	-0.105	-0.096	-0.188	-0.029	0.111	0.204	-0.038
	C4	Primary BAs	Secondary BAs	Unconjugated BAs	Conjugated BAs	Total BAs	Primary / secondary BAs	age	BMI	systolic BP	diastolic BP	glucose	HbA1c	ASAT	ALAT	GFR mayo	total cholesterol	LDL-cholesterol	HDL-cholesterol	non-HDL-cholesterol	triglycerides	waist circumference	HOMA-IR

Statistically significant correlations are printed in bold face (*: p≤0.05; ** p≤0.01).

Most of the BAs were highly correlated with each other. Additional statistically significant correlations were found to exist between secondary BAs and HbA1c (r = 0.166; p = 0.043) or TG (r = 0.181; p = 0.027), between unconjugated BAs and HbA1c (r = 0.203; p = 0.013), TG (r = 0.201; p = 0.014) or HOMA-IR (r = 0.223; p = 0.008) and between total BAs and HbA1c (r = 0.168; p = 0.041), TG (r = 0.177; p = 0.031) or HOMA-IR (r = 0.173; p = 0.040). The ratio primary/secondary BAs did not show any statistically significant correlations with C4, unconjugated BAs, conjugated BAs or any of the parameters characterizing glucose or lipid metabolism.

Because the components of the MetS were also highly correlated with each other, we performed a multiple linear regression analysis to unravel independent determinants of C4 levels as well as the role of C4 as an independent determinant of the other variables. For these tests we included C4, TG, HOMA-IR, HbA1c, non-HDL-C as well as BMI as either the dependent or the determinant variables (Table 7). We did not consider waist circumference, glucose, total or LDL-C because these variables were redundant and correlated less with C4 than BMI, HbA1c, and non-HDL-C, respectively. In this multiple regression analysis, only TG and BMI evolved as significant independent determinants of C4 (adjusted R^2^ = 0.145; p = 7.26E-06). When BMI was set as the dependent variable, it was significantly and independently associated with C4 levels as well as with HOMA-IR (adjusted R^2^ = 0.227; p = 7.03E-09). No other MetS and T2DM parameter was independently determined by C4 (Table 7).

**Table 7:** Results of the multiple regression analysis showing the standardized coefficients for each determinant variable which significantly contributed to a given model.

	Dependant variables					
Determinant variables	C4	Triglycerides	BMI	Non-HDL-C	HOMA-IR	HbA1c
C4	-	n.s.	0.160	n.s.	n.s.	n.s.
Triglycerides	0.295	-	n.s.	0.604	0.134	n.s.
BMI	0.223	n.s.	-	n.s.	0.321	n.s.
Non-HDL-C	n.s.	0.584	n.s.	-	n.s.	−0.145
HOMA-IR	n.s.	0.318	0.422	−0.184	-	0.502
HbA1c	n.s.	n.s.	n.s.	−0.192	0.414	-
Adjusted R-square of model	0.145	0.380	0.227	0.380	0.383	0.284
Significance of model	7.26E-06	1.78E-15	7.03E-09	8.04E-15	5.67E-15	3.53E-11

The variable inclusion criterion was set to p≤0.05.

### Logistic regression and principal component analysis of associations between C4 and MetS

Based on the results of the univariate analyses and correlations described in Tables 3, 5 and 6, logistic regression and principal component analyses were performed to test whether C4 shows a statistically independent association with the MetS. To this end we compared data of MetS and T2DM free controls (N = 49) with combined data from patients with MetS or T2DM (N = 100).

Logistic regression analysis was used to determine whether plasma levels of C4 are associated with MetS and T2DM independently of those components of the MetS which showed the highest correlations with C4 (Table 6), namely TG, HbA1c, HOMA-IR, non-HDL-C and BMI. Upon both forward and backward stepwise regression analysis, the association of C4 with MetS/T2DM was lost, whereas TG and HbA1c remained significantly associated with MetS/T2DM in either model (classification Table: 87%, Nagelkerke R^2^ = 0.639). These results indicate that C4 is not independently associated with MetS and T2DM.

**Figure 3:**
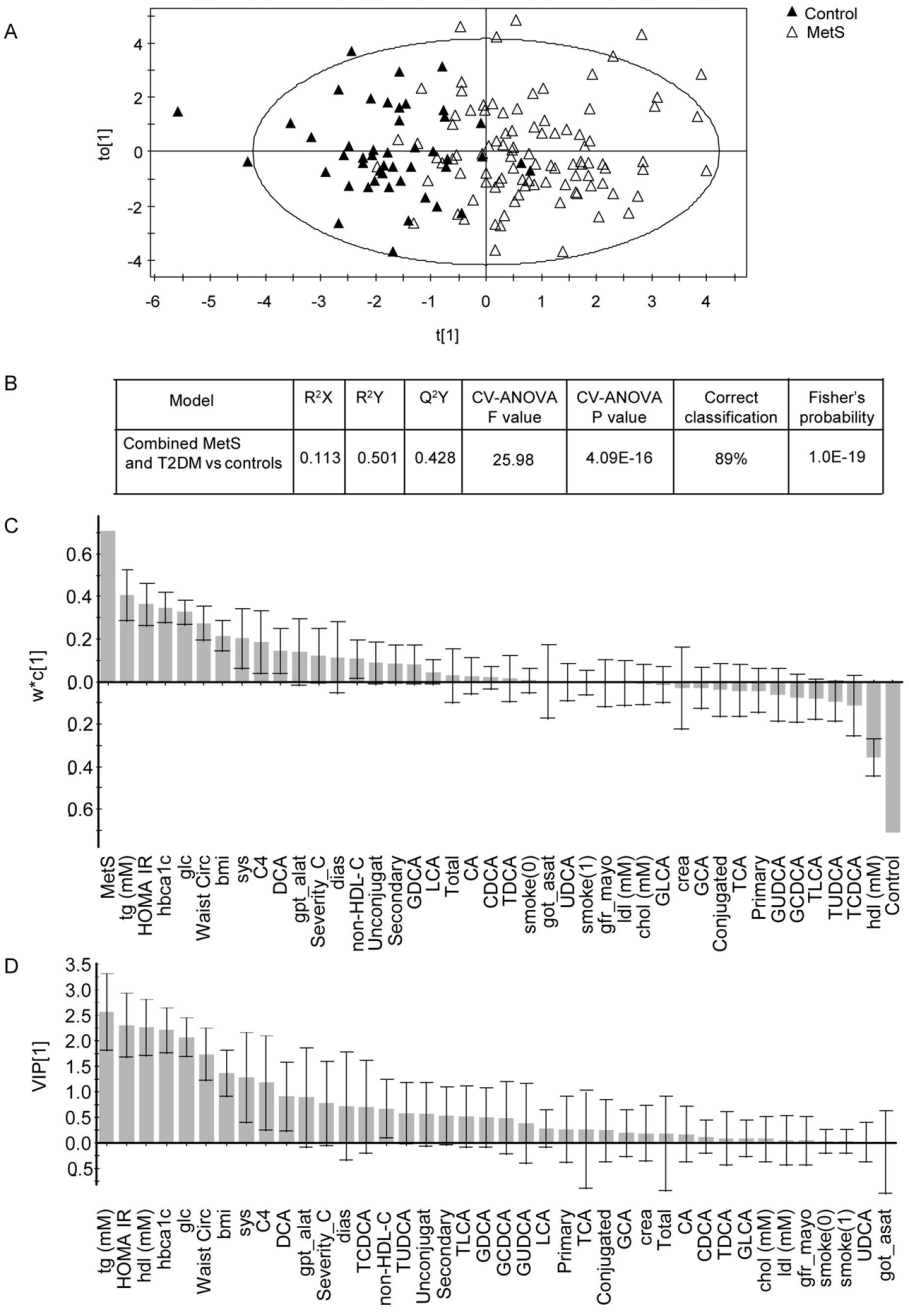
OPLS-DA of combined MetS and T2DM patients (N = 100) versus MetS and T2DM free controls (N = 49). A) Score plots showing individual measurements (black triangles: controls; white triangles: MetS and T2DM patients). B) Model evaluation: R^2^X, R^2^Y and Q^2^Y represent the amount of explained X-variation, Y-variation and predicted Y-variation respectively. Cross validation (CV) is evaluated using the CV-ANOVA p-value, which represents the significance of the predicted Y-variation for the given F value. Correct classification shows the percentage of values classified correctly for the entire group according to the model and Fisher's probability represents the likelihood of obtaining the same classification result by chance. C) Loading column plots with the weights representing the contribution of each variable to the model component scores. D) Variable importance of the projection (VIP) coefficient plot indicating which variables are important in explaining both the X- and the Y-data. Error bars show 95% confidence intervals for the calculated weights and importance.

Figure 3 shows the results of the OPLS-DA. After reducing the dimensions of the data to a single principal component and an orthogonal component, the two groups showed a clear tendency for separation, however complete separation was not achieved (Figure 3a). According to the model evaluation, R^2^Y (percent of the model fitting the data) represented 50% and Q^2^Y (goodness of prediction) represented 43%. The loading plots, which show the weights of the individual variables within the model and hence their contribution to the disease state, revealed that TG, HDL-C (inversely), HOMA-IR, HbA1c, glucose, waist circumference, BMI and systolic blood pressure contributed most strongly to the model (95% CI>0) (Figure 3c). Among the BA parameters, C4 was the strongest contributor to the model, followed by the secondary BA DCA. All other parameters (including all BAs except DCA) had a 95% CI beyond zero and therefore they did not significantly contribute to the model. The variable importance for the projection (VIP) plot shows the contribution of each variable to the variation in both the X space and the Y space (hence its correlation with other variables and the control or disease state). A coefficient value >1 means that the variable is “important”. In our model, the VIP plot showed that TG, HOMA-IR, HDL-C, HbA1c, glucose and waist circumference had the highest VIP coefficients (Figure 3d). BMI, systolic blood pressure, C4 and DCA also contributed to the model; however their 95% CI extended below 1.0, so that their contribution to the model must be interpreted with caution. All other parameters did not significantly contribute to the model according to the VIP plot.

Another OPLS-DA model was fitted to the data of the 50 T2DM patients versus the 49 controls without MetS and T2DM (Figure S1). In this case, the separation between the two groups became clearer although some overlap was still present. R^2^Y and Q^2^Y represented 69% and 58% respectively. In the loading plot, the variables contributing most strongly to the T2DM model were the same as in the previous model but with HbA1c, glucose and HOMA-IR coming first as expected. A noteworthy difference in this model was that C4 contributed more strongly to the model than did systolic blood pressure.

## Discussion

This study provides the as yet most comprehensive data on the diurnal intraindividual variation of serum concentrations of 15 BA species and their biosynthetic precursor C4 as well as on their associations with CAD, T2DM, and non-diabetic MetS.

### Diurnal variation of BAs and C4

During a 24-hours cycle, all BA species and C4 varied considerably in their serum concentrations with variation coefficients between 42% and 72% for C4 and reaching up to 190% for TDCA in subject A (Table 1). In addition, although we studied only four probands, there is considerable interindividual variation in both the timing and quantity of peak and trough levels of BA or C4 in serum (Figure 2). Despite this limitation, we draw some conclusions on both the physiology behind the intraindividual variation and the consequences for the clinical or epidemiological validation of BA and C4 as biomarkers.

The reasons for the high degree of intraindividual variation appear to differ for the various BAs and C4: conjugated BAs appear to be altered by the prandial state whereas unconjugated BAs seem to undergo diurnal changes independently of food intake. The strong prandial effect on total and conjugated BAs was observed already previously [45], [52], [53], [54], whereas the diurnal changes of unconjugated BAs independently of food intake have not yet been described.

We, like other authors [53], [54], found that serum levels of conjugated BAs are strongly influenced by food intake and peak one to two hours after food intake (Figures 1b and c, Figure 2). However, since serum levels of conjugated BAs already increased before breakfast and decrease more slowly in the night after supper than in the afternoon after lunch we cannot exclude additional non-prandial effects on serum levels of conjugated BAs. Interestingly in our experiment all ten conjugated BA species were found to increase in parallel. This is in contrast to other reports where CDCA and DCA concentrations were described to increase more rapidly than CA concentrations, possibly due to passive reabsorption of the dihydroxylated BAs in the proximal intestine as opposed to active absorption of CA in the ileum [52], [53].

Unconjugated BA concentrations increased dramatically during the night and early morning (Figures 1a and 2a). Since supper was taken at least 5 hours earlier at 20h15 and since other meals caused no or much less prominent increases of unconjugated BAs, this phenomenon appears to reflect diurnal biorhythm rather than postprandial effects. However, our findings are in contrast to those of other authors who observed maximal serum concentrations of unconjugated BAs between breakfast and dinner with little changes between midnight and breakfast [55]. They suggested the existence of a pool of unconjugated BAs in the intestine, which is reabsorbed as a consequence of increased intestinal motility following breakfast intake. We have no explanation for the discrepant findings except that the sleep-wake phase may have been shifted in that study compared to ours. Interestingly, the maxima in the concentrations of unconjugated BAs observed by us occur close to the daytime at which BA biosynthesis was previously found to be maximal, namely at 06h00 or 09h00 [46], [56]. It is hence tempting to hypothesize that the peak serum concentration of unconjugated BAs reflects peak BA biosynthesis. However, as discussed before, the unconjugated BAs recovered in the bloodstream are of intestinal rather than direct hepatic origin. Moreover, one would expect only the primary BAs CA and CDCA to be elevated if the peak observed was due to BA biosynthesis. However DCA levels peaked to the same degree as did CDCA levels (but less than CA levels). Therefore and because serum levels of the biosynthetic precursor C4 peaked considerably earlier (Figure 2d), we consider it as unlikely that the peak of unconjugated BAs observed during the night reflects increased BA biosynthesis.

The C4 profile showed two peaks, one between 19h00 and 01h00 and another between 11h00 and 15h00 (Figure 2D). Our findings are in accordance with those of a previous study in which also two peaks at 21h00 and 13h00 were observed [45] but only in partial agreement with another study in which only one peak was observed at approximately 13h00 [57]. Our observation is also in accordance with the two studies which measured radiolabelled CO_2_ in the breath as a marker of BA biosynthesis. In the classical pathway of BA biosynthesis, which accounts for the majority the biosynthesis, the shortening of the side chain and subsequent release of radiolabelled propionic acid takes place at a relatively late stage in the pathway [58]. This would explain the several hours delay between the peak observed for C4 and the peak of labelled CO_2_ in the breath.

In conclusion, because of chronotropic and prandial effects, unconjugated and conjugated BAs as well as C4 show a very high degree of intraindividual variation, which makes their clinical use as biomarkers difficult. The need to be consistent regarding blood sampling time and fasting state was already mentioned in 1980 [59] and this issue is still ongoing since total BAs are commonly measured as a routine test for the diagnosis of hepatobiliary diseases. Conjugated BA levels can be controlled rather easily by the use of fasting blood only. However unconjugated BAs and C4 are more difficult to control since their acrophase is early in the morning and because already the four probands of our study showed considerable interindividual variations in the time course of unconjugated BA and C4. For example it is very likely that many fasting blood samples drawn typically between 08h00 and 10h00 as in our case-control study will contain levels of unconjugated BAs which have not returned to baseline at the time of sampling.

### Association of BAs and C4 with CAD, T2DM and MetS

In our study none of the BA parameters was associated with CAD upon univariate statistical analyses, therefore we did not perform further analyses with the CAD classification. By contrast, both MetS and T2DM were associated with similarly elevated plasma levels of C4. This finding appears to be in agreement with previous studies which reported increased BA synthesis in diabetic patients, at least before insulin treatment [39]–[41], [60]. Upon multivariate analysis of our data however, the association of C4 with T2DM and MetS was lost, mainly because of the dependence of C4 levels of TG and BMI. In the orthogonal partial least square-discriminant analysis as well, C4 was a weaker contributor to MetS than its canonical components. We, like others, also could not detect statistically significant associations of other serum markers of BA metabolism with T2DM or MetS upon univariate analyses, including those which were previously associated with T2DM, namely total CDCA or GCDCA [41], [61], primary to secondary BAs ratio [44] and CA to CDCA ratio [45].

The weaker as well as statistically not independent association of C4 (and probably the absent associations of BAs) with MetS or T2DM must be interpreted in the light of the very large interindividual variation of BAs and C4 serum levels. The coefficient of variation for C4 within the individual cohorts ranged from 62% to 99% compared to 33% to 78% for TG, which otherwise is the most variable component of the MetS. One important reason for this large variation is the diurnal variation of C4 and BA levels which we described and discussed before. This large intraindividual variation translates into even larger interindividual variation because not all participants are synchronized and it is further increased by additional influencing factors. Serum levels of BAs are even more strongly influenced by internal factors than C4, because they undergo an extensive enterohepatic circulation. Thus the lack of independent association of C4 and the lack of any statistically significant association of the BAs with MetS and T2DM does not contradict the pathogenic links between BA metabolism and MetS or T2DM, for which many lines of evidence have been produced by animal experiments and intervention studies in humans [62], [63].

Interestingly, C4 showed several positive and statistically significant correlations with single components of the MetS, namely BMI, TG, glucose, HbA1c, and HOMA-IR. Some of these variables also showed statistically significant positive correlations with other parameters of BA metabolism, namely secondary, unconjugated and total BAs, but none was as strong as those with C4, probably also because of the biological reasons indicated before. Only the positive correlation between C4 and TG is in agreement with the hypertriglyceridemic effects of BA sequestration or inborn defects of intestinal BA reabsorption [64]. By contrast, the positive and significant correlations of C4 and the BAs with non-HDL-C, glucose, HbA1c and HOMA-IR are in contrast to known regulatory functions of BAs as well as several previous findings made by intervention studies in men or mice: BAs activate the nuclear hormone receptor FXR and thereby repress several lipogenic and gluconeogenic target genes and hence lower plasma levels of cholesterol, TG and glucose [65]. Most recently BAs were also found to activate the G-protein coupled receptor TGR5 and thereby exert anti-obesity as well as insulin sensitizing effects [66]. The cholesterol lowering and euglycemic effects of treatment with BA sequestrants [7], [60], which causes a compensatory increase of BA synthesis by 40% [60], are also in opposition with the positive correlations that we observed for C4. Moreover, plasma concentrations of BA were found significantly increased in patients with prior gastric bypass surgery as compared to both overweight and obese controls and to correlate negatively both with fasting triglycerides and 2 h-post meal-glucose [67].

A possible explanation for our, at first sight counterintuitive observations is provided by the results of our multiple regression analyses.

On the one hand and interestingly, C4 levels were not found to be an independent determinant of the components of MetS and T2DM except for BMI suggesting that bile acids may affect body weight gain. In this regard it is interesting to note, that FXR is expressed in murine adipocytes where it appears to promote adipocyte differentiation as well as lipogenesis and to decrease lipolysis [68]. In agreement with a physiological role of these in vitro observations, in a mouse model of obesity, FXR deficiency rather than FXR stimulation attenuated body weight gain and improved insulin sensitivity by extrahepatic FXR effects [69].

On the other hand and even more importantly, BMI and TG were identified as independent determinants of C4 serum levels. This unexpected and as yet unknown direction of multivariate correlations suggests that the synthesis of BAs is influenced by triglyceride metabolism and adipose tissue. As yet this direction of the possibly mutual relationships between BA synthesis and several components of the MetS has not been much investigated by experiments. For example, patients with gallstones, which occur at an increased frequency in patients with MetS, show increased production of both TG and BAs [70]. Finally, one must consider the possibility that BA synthesis, triglyceride metabolism and adipose tissue are not only causally linked with each other but regulated in parallel by another factor.

Our study has several limitations. The problems of diurnal variation and the resulting large intra- and interindividual variations of C4 and BAs have been described and discussed before. In addition we cannot exclude that the young and healthy volunteers of our pilot study differ from the older and metabolically compromised patients of our clinical study in the degree and time course of intraindividual variation. In view of the large interindividual variation in C4 and BA levels, our clinical study also suffers from a relatively small study size (N = 150), although to the best of our knowledge, it is as yet the largest study investigating the associations of CAD, T2DM and MetS with BAs and C4. In addition, basal fasting bile acid levels are low and may not help to discriminate physiological and pathological bile acid metabolism between the different patient and control cohorts of our study. Provocation by standardized test meals or treatment with bile acid sequestrants as in some other small studies [60], [61] may have helped to identify differences in bile acids. However this kind of provocation studies needs long term intervention with standardized meals or bile acid sequestration which are not feasible in the clinical setting of our study. A third limitation is the medication of many patients with statins or anti-diabetics which may influence BA synthesis and metabolism, although we did not see any statistically significant differences between consumers and non-consumers of these drugs. Finally, we cannot firmly exclude the asymptomatic presence of liver or bile duct diseases in our patients since no ultrasound examinations have been performed. However, none of the patients presented with strongly elevated liver enzymes. Therefore and because bile acids are considered as very sensitive markers of cholestatic liver disease but were in the normal range in ou patients, we assume that none of the patients presented with relevant liver or bile duct disease.

In conclusion, despite the considerable intra- and interindividual variation of BA and C4 serum levels, patients with MetS and T2DM present with significantly increased plasma levels of C4, a biomarker of BA synthesis. This association is confounded by positive correlations of C4 with TG and BMI. These data suggest that BAs are not only influencing lipid and glucose metabolism as well as adiposity but also that triglyceride metabolism and adipose tissue reciprocally influence BA synthesis.

## Supporting Information

Figure S1 **OPLS-DA of T2DM patients (N = 50) versus MetS and T2DM free controls (N = 49).** A) Score plots showing individual measurements (black triangles: controls; white triangles: MetS and T2DM patients). B) Model evaluation: R^2^X, R^2^Y and Q^2^Y represent the amount of explained X-variation, Y-variation and predicted Y-variation respectively. Cross validation (CV) is evaluated using the CV-ANOVA p-value, which represents the significance of the predicted Y-variation for the given F value. Correct classification shows the percentage of values classified correctly for the entire group according to the model and Fisher's probability represents the likelihood of obtaining the same classification result by chance. C) Loading column plots with the weights representing the contribution of each variable to the model component scores. D) Variable importance of the projection (VIP) coefficient plot indicating which variables are important in explaining both the X- and the Y-data. Error bars show 95% confidence intervals for the calculated weights and importance.
(TIF)

Table S1 Spearman correlation matrix (2-tailed) of bile acids and C4 with age. No significant correlations were observed.
(DOC)

Table S2 Bile acid and C4 levels in patients according to the gender and the intake of medication. ^a^Mann-Whitney U test.
(DOC)
