# Supplementary material for: Bile Acid Metabolites in Serum: Intraindividual Variation and Associations with Coronary Heart Disease, Metabolic Syndrome and Diabetes Mellitus
Source: PLoS One. 2011 Nov 14;6(11):e25006. doi: 10.1371/journal.pone.0025006 (PMC3215718; doi:10.1371/journal.pone.0025006)
Supplement: Table S1 — Spearman correlation matrix (2-tailed) of bile acids and C4 with age. No significant correlations were observed. (DOC) [file pone.0025006.s002.doc]

**Table S1**

|  | Healthy cohort (CAD:no/MetS:no/T2DM:no) (N = 24) | Entire cohort (N = 149) |
| --- | --- | --- |
|  | Age | Age |
| C4 | -0.006 | 0.071 |
| Primary BAs | 0.048 | -0.028 |
| Secondary BAs | -0.126 | -0.006 |
| Unconjugated BAs | -0.038 | -0.030 |
| Conjugated BAs | -0.060 | 0.022 |
| Total BAs | 0.035 | 0.006 |

Spearman correlation matrix (2-tailed) of bile acids and C4 with age. No significant correlations were observed.
