# Supplementary material for: Bile Acid Metabolites in Serum: Intraindividual Variation and Associations with Coronary Heart Disease, Metabolic Syndrome and Diabetes Mellitus
Source: PLoS One. 2011 Nov 14;6(11):e25006. doi: 10.1371/journal.pone.0025006 (PMC3215718; doi:10.1371/journal.pone.0025006)
Supplement: Table S2 — Bile acid and C4 levels in patients according to the gender and the intake of medication. aMann-Whitney U test. (DOC) [file pone.0025006.s003.doc]

**Table S2: Bile acid and C4 serum** levels in patients according to the gender and the intake of medication

|  | **C4** | **Primary bile acids** | **Secondary bile acids** | **Unconjugated bile acids** | **Conjugated bile acids** | **Total bile acids** |
| --- | --- | --- | --- | --- | --- | --- |
|  | median (range) [μmol/L] | median (range) [μmol/L] | median (range) [μmol/L] | median (range) [μmol/L] | median (range) [μmol/L] | median (range) [μmol/L] |
| **Healthy cohort (CAD:no/MetS:no/T2DM:no) (N = 24)** |  |  |  |  |  |  |
| Female (N = 14) | 0.029 (0.007-0.092) | 2.15 (0.411-8.02) | 1.36 (0.511-3.13) | 1.07 (0.473-8.20) | 1.57 (0.517-5.49) | 3.45 (1.00-10.1) |
| Male (N = 10) | 0.042 (0.018-0.146) | 1.74 (0.154-13.0) | 1.33 (0.073-8.93) | 1.03 (0.112-5.11) | 1.45 (0.115-21.1) | 3.29 (0.227-22.0) |
| p-value^a^ | 0.198 | 0.447 | 0.953 | 0.639 | 0.558 | 0.598 |
| **Entire cohort (N = 149)** |  |  |  |  |  |  |
| Female (N = 89) | 0.040 (0.004-0.260) | 1.64 (0.299-8.02) | 1.21 (0.074-5.30) | 1.17 (0.165-8.20) | 1.26 (0.343-6.50) | 2.85 (0.831-10.1) |
| Male (N = 60) | 0.053 (0.003-0.267) | 1.25 (0.154-13.0) | 1.09 (0.073-8.93) | 1.00 (0.112-8.26) | 1.09 (0.115-21.1) | 2.60 (0.227-22.0) |
| p-value^a^ | 0.397 | 0.217 | 0.665 | 0.270 | 0.455 | 0.430 |
| **MetS and T2DM free controls (N = 49)** |  |  |  |  |  |  |
| Statins: - (N = 32) | 0.029 (0.004-0.146) | 1.52 (0.154-13.0) | 1.13 (0.073-8.93) | 0.967 (0.112-8.20) | 1.41 (0.115-21.1) | 2.74 (0.227-22.0) |
| Statins: + (N = 17) | 0.029 (0.007-0.114) | 1.89 (0.319-4.37) | 1.10 (0.286-2.81) | 1.15 (0.429-2.89) | 1.77 (0.223-5.18) | 3.15 (0.712-6.08) |
| p-value^a^ | 0.475 | 0.801 | 0.785 | 0.644 | 0.629 | 0.883 |
| **MetS patients (N = 50)** |  |  |  |  |  |  |
| Statins: - (N = 30) | 0.060 (0.009-0.143) | 1.29 (0.299-5.16) | 0.956 (0.273-3.51) | 1.13 (0.333-3.94) | 0.911 (0.345-5.29) | 2.40 (1.14-7.32) |
| Statins: + (N = 20) | 0.038 (0.006-0.258) | 1.33 (0.329-9.56) | 1.08 (0.241-8.01) | 1.09 (0.287-5.52) | 1.24 (0.343-16.4) | 2.58 (0.884-17.6) |
| p-value^a^ | 0.060 | 0.828 | 0.722 | 0.843 | 0.859 | 0.859 |
| **T2DM patients (N = 50)** |  |  |  |  |  |  |
| Statins: - (N = 17) | 0.059 (0.023-0.260) | 1.78 (0.207-5.33) | 1.49 (0.535-4.40) | 1.48 (0.541-4.82) | 1.51 (0.172-6.50) | 3.51 (0.867-9.73) |
| Statins: + (N = 33) | 0.050 (0.003-0.267) | 1.64 (0.277-9.27) | 1.29 (0.115-5.30) | 0.995 (0.195-8.26) | 1.15 (0.362-4.92) | 3.09 (0.831-9.40) |
| p-value^a^ | 0.193 | 0.602 | 0.256 | 0.215 | 0.927 | 0.430 |
| **CAD free controls (N = 74)** |  |  |  |  |  |  |
| Statins: - (N = 48) | 0.052 (0.007-0.260) | 1.76 (0.154-13.0) | 1.34 (0.073-8.93) | 1.29 (0.112-8.20) | 1.44 (0.115-21.1) | 3.31 (0.227-22.0) |
| Statins: + (N = 26) | 0.045 (0.006-0.138) | 1.62 (0.411-9.56) | 1.31 (0.374-8.01) | 1.17 (0.274-6.03) | 0.993 (0.343-16.4) | 3.21 (0.916-17.6) |
| p-value^a^ | 0.141 | 0.651 | 0.448 | 0.726 | 0.402 | 0.548 |
| **CAD patients (N = 75)** |  |  |  |  |  |  |
| Statins: - (N = 31) | 0.033 (0.004-0.143) | 1.26 (0.207-4.36) | 0.925 (0.074-4.06) | 1.00 (0.165-5.00) | 0.939 (0.222-6.50) | 2.16 (0.777-7.71) |
| Statins: + (N = 44) | 0.037 (0.003-0.267) | 1.61 (0.277-9.27) | 1.09 (0.115-5.30) | 1.02 (0.195-8.26) | 1.23 (0.223-5.18) | 2.78 (0.712-9.40) |
| p-value^a^ | 0.863 | 0.172 | 0.613 | 0.872 | 0.093 | 0.186 |
| **T2DM patients (N = 50)** |  |  |  |  |  |  |
| Insulin: - (N = 35) | 0.055 (0.003-0.267) | 1.78 (0.207-5.33) | 1.34 (0.374-5.30) | 1.21 (0.274-6.94) | 1.85 (0.298-6.50) | 3.51 (0.867-9.73) |
| Insulin: + (N = 15) | 0.042 (0.013-0.179) | 1.29 (0.291-9.27) | 1.31 (0.115-3.00) | 1.49 (0.195-8.26) | 0.859 (0.172-3.63) | 2.49 (0.831-9.40) |
| p-value^a^ | 0.216 | 0.172 | 0.415 | 0.695 | 0.105 | 0.335 |
| **T2DM patients (N = 50)** |  |  |  |  |  |  |
| Oral antidiabetics: - (N = 25) | 0.044 (0.003-0.180) | 1.48 (0.207-5.33) | 1.21 (0.195-4.40) | 1.17 (0.195-6.03) | 0.859 (0.298-6.50) | 2.71 (0.867-9.73) |
| Oral antidiabetics: + (N = 25) | 0.056 (0.014-0.267) | 1.71 (0.291-9.27) | 1.53 (0.115-5.30) | 1.48 (0.395-8.26) | 1.38 (0.172-4.92) | 4.06 (0.831-9.40) |
| p-value^a^ | 0.327 | 0.265 | 0.204 | 0.299 | 0.114 | 0.082 |

^a^Mann-Whitney U test
